# Supplementary figures and images for: Non-canonical regulation of the reactivation of an oncogenic herpesvirus by the OTUD4-USP7 deubiquitinases
Source: PLoS Pathog. 2024 Jan 12;20(1):e1011943. doi: 10.1371/journal.ppat.1011943 (PMC10810452; doi:10.1371/journal.ppat.1011943)

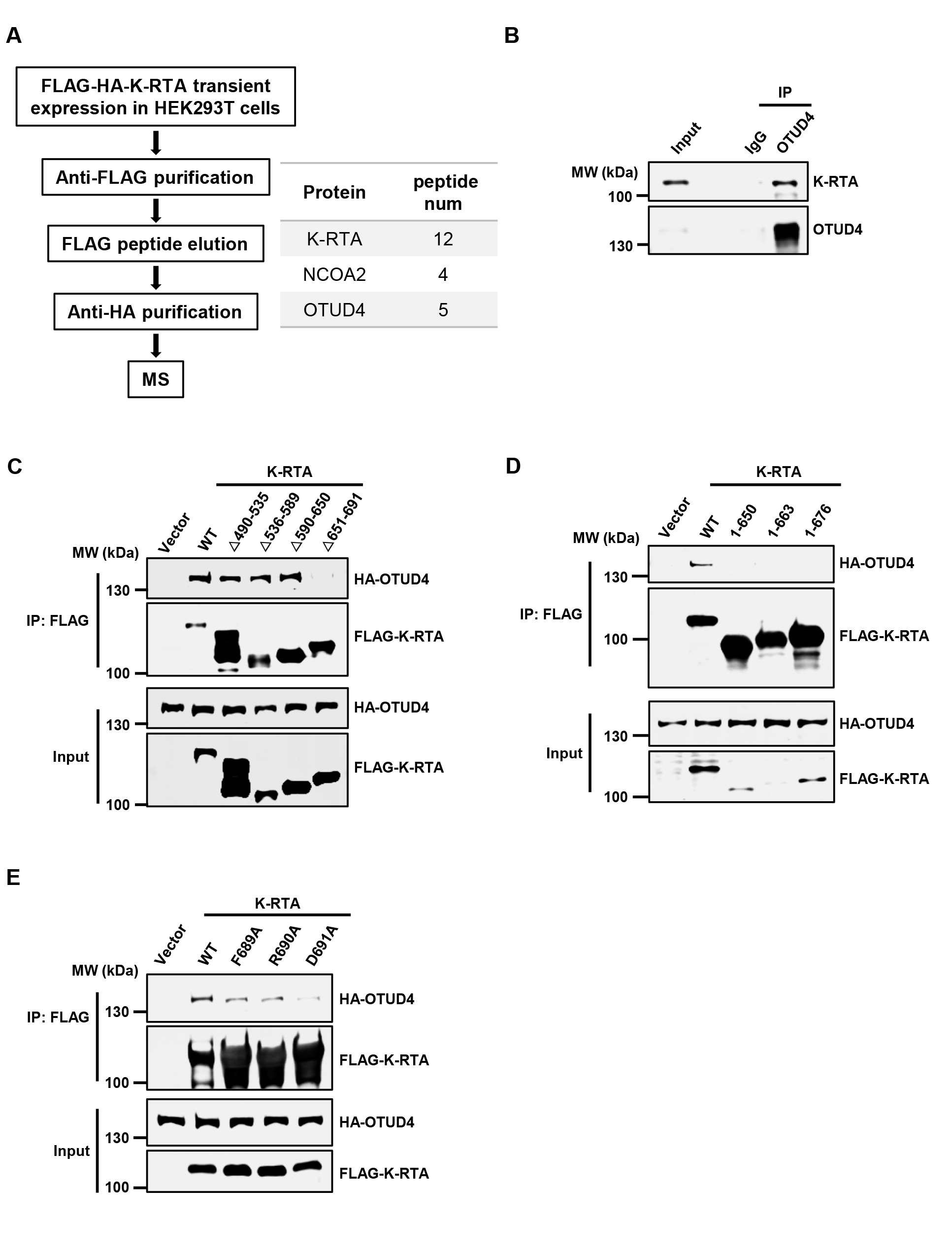

Supplement: S1 Fig — (A) Affinity purification followed by mass spectrometry analysis to identify K-RTA binding proteins. The number of identified peptides corresponding to K-RTA, NCOA2, and OTUD4 was summarized. (B) BCBL1-Tet-K-RTA cells were induced with Dox (1 μg/ml) and sodium butyrate (0.5 mM) for 48 h, and co-immunoprecipitation and immunoblotting were performed with the indicated antibodies. (C-E) HEK293T cells were transfected with the indicated plasmids, and WCLs were collected for immunoprecipitation with anti-FLAG affinity agarose, followed by immunoblotting. The interaction between K-RTA truncations, including K-RTAΔ490–535, K-RTAΔ536–589, K-RTAΔ590–650, and K-RTAΔ651–691, and OTUD4 was assessed by co-immunoprecipitation in HEK293T cells (C). The interaction between K-RTA truncations, including K-RTA (1–650), K-RTA (1–663), and K-RTA (1–676), and OTUD4 was assessed by co-immunoprecipitation (D). The interaction between K-RTA point mutations, including K-RTA(F689A), K-RTA(R690A), and K-RTA(D691A), and OTUD4 was assessed by co-immunoprecipitation (E). (TIF) [file ppat.1011943.s001.tif]

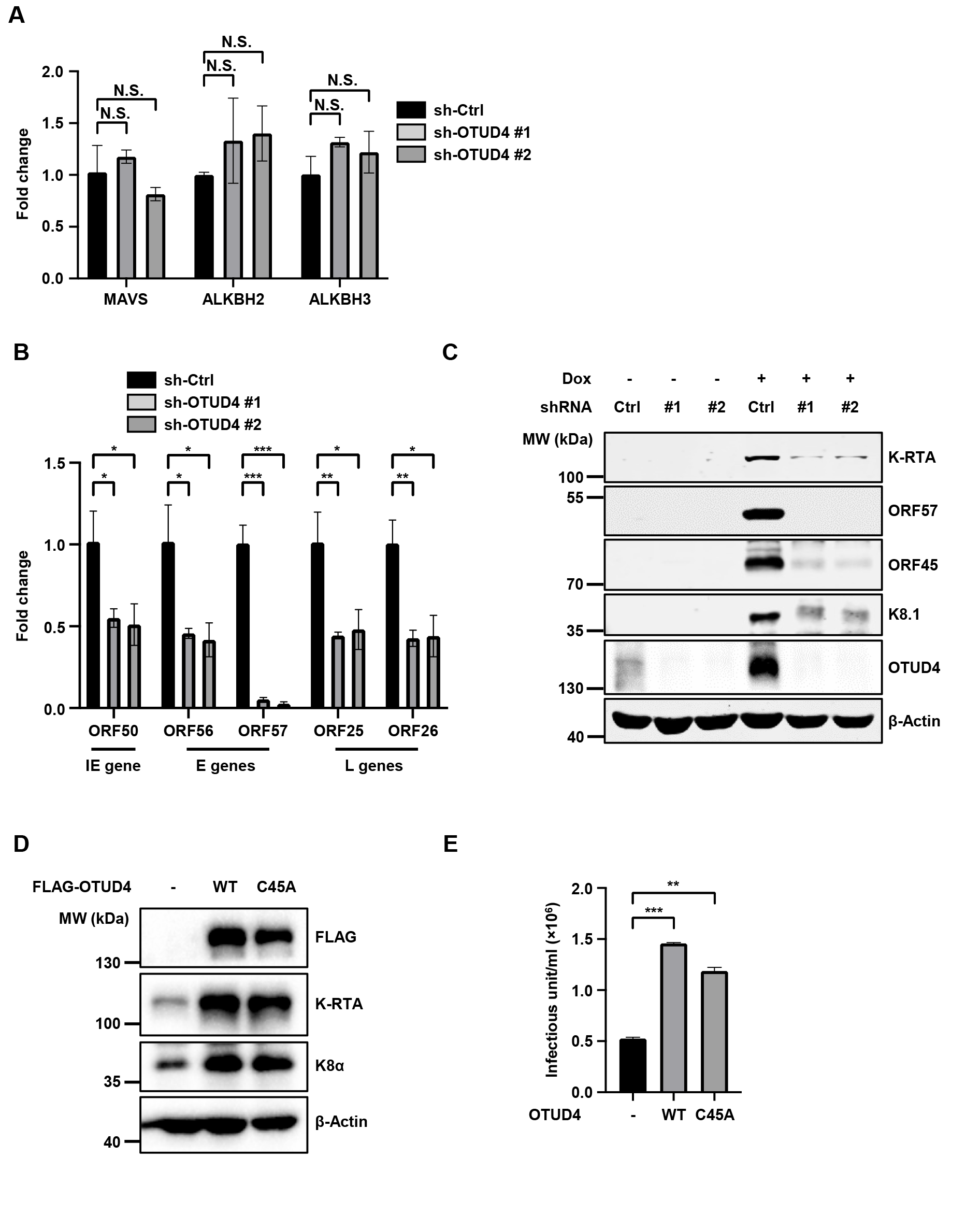

Supplement: S2 Fig — (A) SLK.iBAC-GFP cells stably transduced with sh-Ctrl or sh-OTUD4 were induced with Dox (1 μg/ml) for 48 h, and the expression of the indicated gene was quantified by RT-qPCR. (B) BCBL1-Tet-K-RTA cells stably transduced with sh-Ctrl or sh-OTUD4 were induced with Dox (1 μg/ml) for 48 h, followed by quantification of viral gene expression by RT-qPCR. (C) Immunoblot of BCBL1-Tet-K-RTA cells as described in S2B Fig. (D) SLK.iBAC stably expressing OTUD4 WT or the C45A mutant was induced with Dox (1 μg/ml) for 48 h, followed by immunoblotting analysis. (E) SLK.iBAC-GFP stable cells as described in S2D Fig were induced with Dox (1 μg/ml) and sodium butyrate (0.5 mM) for 48 h. KSHV infectious units in the supernatants were determined. (TIF) [file ppat.1011943.s002.tif]

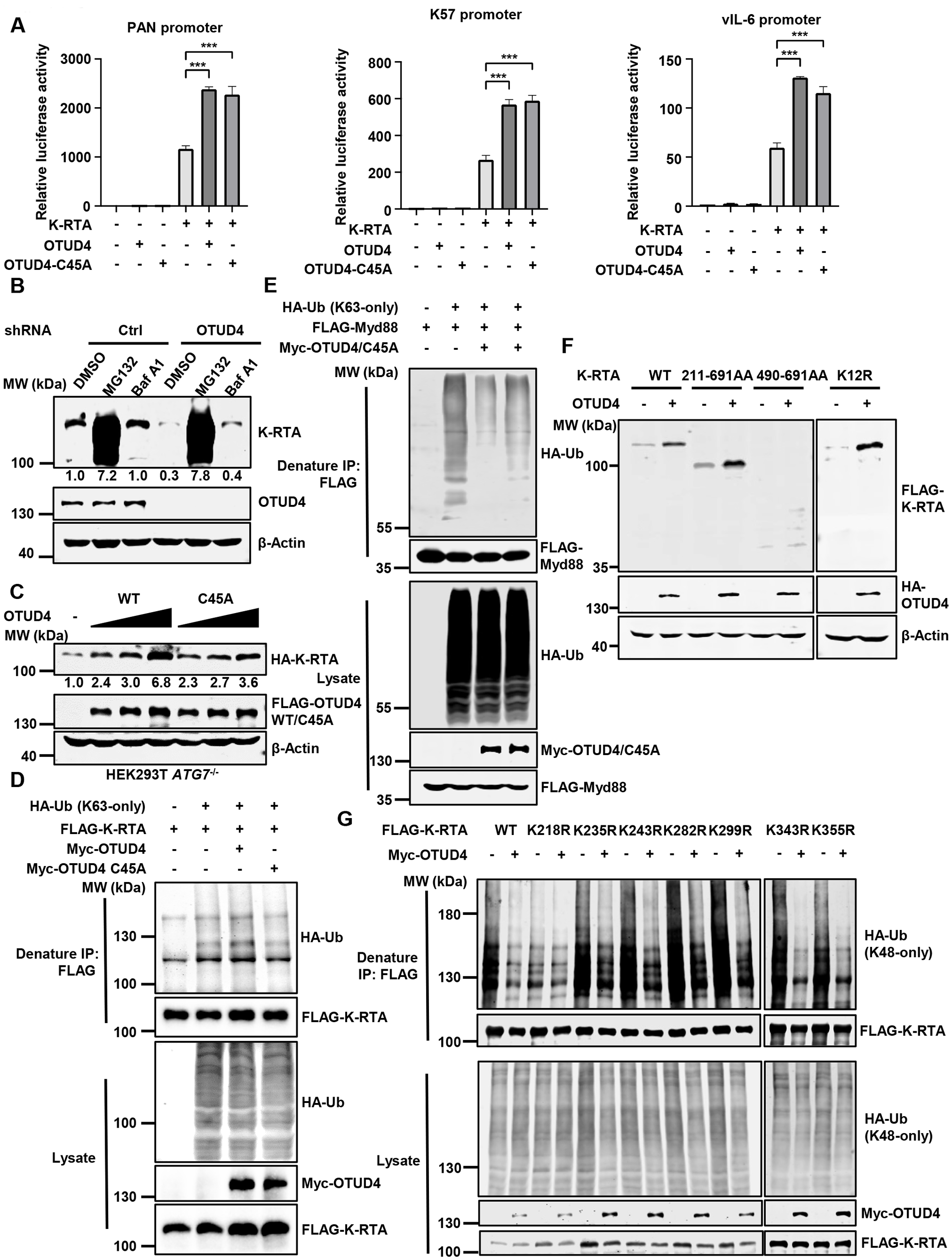

Supplement: S3 Fig — (A) PAN-, K57- or vIL-6-reporter was co-expressed with HA-K-RTA and FLAG-OTUD4 WT or C45A mutant in HEK293T cells. Luciferase activities were determined at 24 h post-transfection. (B) iSLK cells transduced with sh-Ctrl (Scramble) or sh-OTUD4 were treated with DMSO, Baf-A1 (1 mM) or MG132 (10 μM), followed by Dox (0.2 μg/ml) induction for 12 h. WCL were collected and analyzed by immunoblotting. (C) HEK293T cells transduced with sgRNA targeting ATG7 were co-transfected with K-RTA and FLAG-OTUD4/C45A (0, 0.5, 1 or 2 μg), followed by immunoblotting at 24 h post-transfection. (D) HEK293T cells were co-transfected with FLAG-K-RTA, HA-Ub (K63-only) and Myc-OTUD4/C45A, and then treated with MG132 (10 μM). Denatured immunoprecipitation with anti-FLAG affinity agarose was performed, followed by immunoblotting. (E) HEK293T cells were co-transfected with FLAG-Myd88, HA-Ub (K63-only) and Myc-OTUD4/C45A. Denatured immunoprecipitation with anti-FLAG affinity agarose was performed, followed by immunoblotting. (F) HEK293T cells were co-transfected with HA-OTUD4 and FLAG-K-RTA or the indicated mutants, followed by immunoblotting at 24 h post-transfection. (G) FLAG-K-RTA WT or the indicated mutants were co-expressed with HA-Ub (K48-only) in HEK293T cells. Then denatured immunoprecipitation was performed as described in S3D Fig. (TIF) [file ppat.1011943.s003.tif]

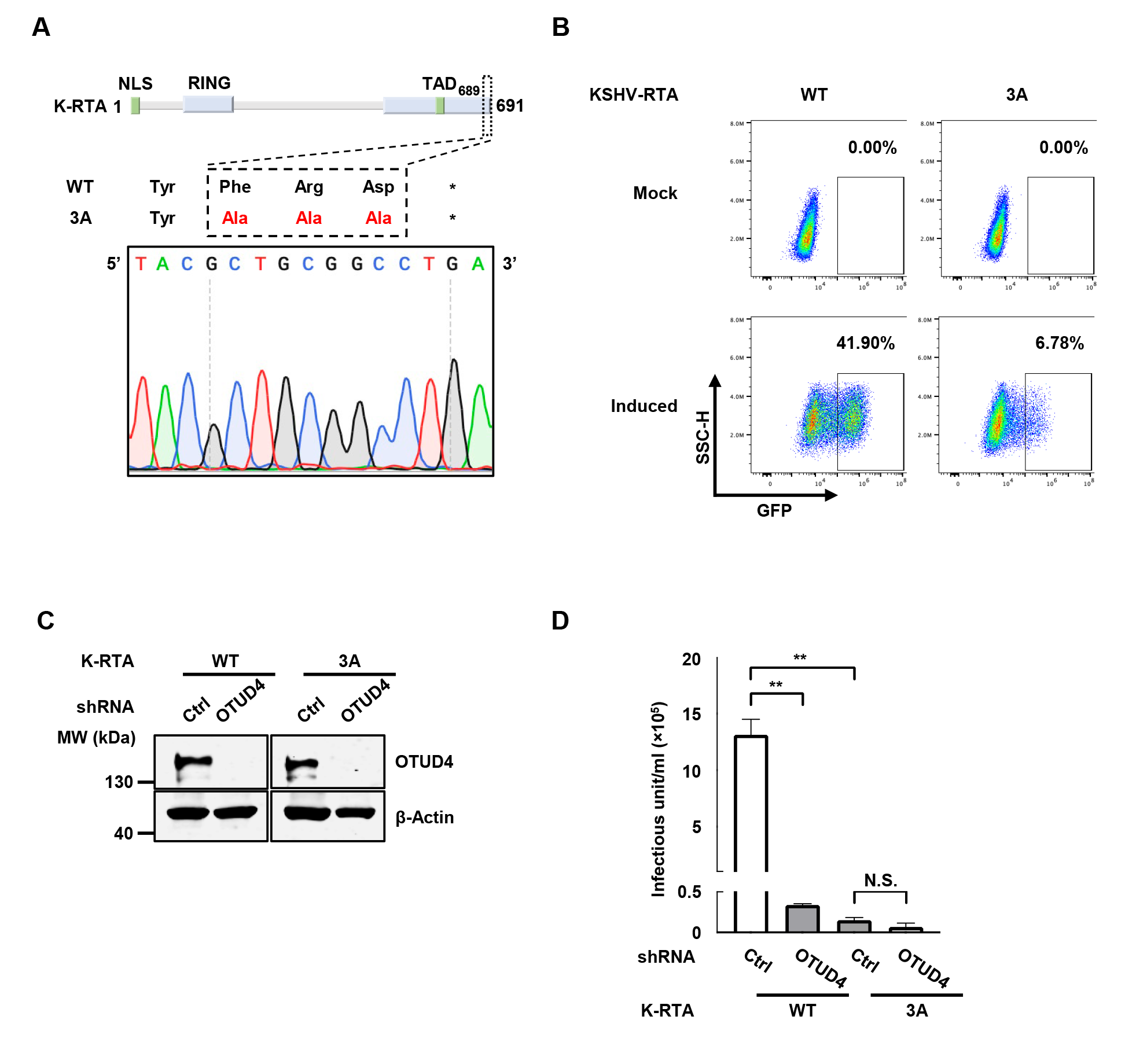

Supplement: S4 Fig — (A) PCR products amplified from the K-RTA locus of KSHV-K-RTA-3A were analyzed by Sanger sequencing. (B) SLK.iBAC-K-RTA-WT or SLK.iBAC-K-RTA-3A cells were induced with Dox (1 μg/ml) and sodium butyrate (0.5 mM) for 48 h. The supernatants containing infectious virion were collected and used to infect HEK293T cells, and the infected cells were analyzed by flow cytometry at 24 h post-infection. (C) SLK.iBAC-K-RTA-WT or SLK.iBAC-K-RTA-3A cells were transduced with sh-Ctrl or sh-OTUD4, and WCLs were analyzed by immunoblotting. (D) SLK.iBAC-K-RTA-WT or SLK.iBAC-K-RTA-3A cells transduced with sh-Ctrl or sh-OTUD4 were induced with Dox (1 μg/ml) and sodium butyrate (0.5 mM) for 48 h, and KSHV infectious units were quantified. (TIF) [file ppat.1011943.s004.tif]

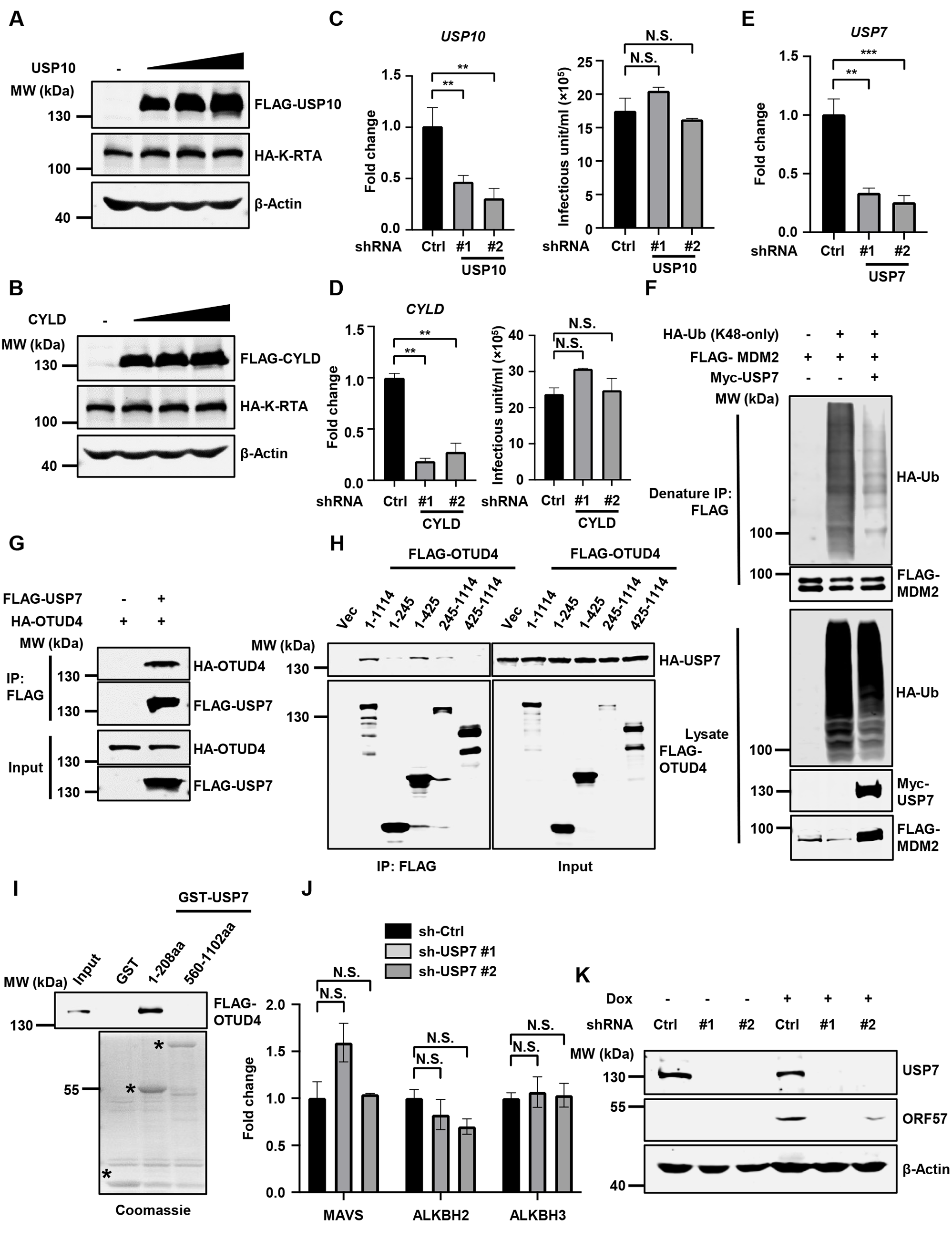

Supplement: S5 Fig — (A-B) HEK293T cells were co-transfected with HA-K-RTA and FLAG-USP10/CYLD (0, 0.5, 1 or 2 μg), and immunoblotting was performed at 24 h post-transfection. (C-D) SLK.iBAC-GFP cells were transduced with sh-Ctrl, sh-USP10 (C) or sh-CYLD (D), and the stable cells were induced with Dox (1 μg/ml) and sodium butyrate (0.5 mM). The expression of the indicated genes was quantified by RT-qPCR, and KSHV infectious units in the supernatants were quantified at 48 h post-induction. (E) SLK.iBAC-GFP cells were transduced with sh-Ctrl or sh-USP7, and the stable cells were induced with Dox (1 μg/ml) and sodium butyrate (0.5 mM) at 48 h post-transduction. The expression of USP7 was quantified by RT-qPCR. (F) HEK293T cells were co-transfected with FLAG-MDM2, HA-Ub (K48-only) and Myc-USP7. Denatured immunoprecipitation with anti-FLAG affinity agarose was performed, followed by immunoblotting. (G) HEK293T cells were co-expressed with FLAG-USP7 and HA-OTUD4, followed by co-immunoprecipitation and immunoblotting at 24 h post-transfection. (H) HEK293T cells were co-expressed with HA-USP7 and FLAG-OTUD4 or the indicated mutants, and WCLs were collected for immunoprecipitation with anti-FLAG affinity agarose at 24 h post-transfection. The input and precipitated samples were analyzed by immunoblotting. (I) GST or GST fusion proteins [GST-USP7 (1-208aa) or GST-USP7 (560-1102aa)] were incubated with FLAG-OTUD4 expressed in HEK293T cells. The binding fractions were analyzed by immunoblotting, and purified GST and GST fusion proteins were visualized by Coomassie Brilliant Blue staining. (J) SLK.iBAC-GFP cells stably transduced with sh-Ctrl or sh-USP7 were induced with Dox (1 μg/ml) for 48 h, and the expression of the indicated gene was quantified by RT-qPCR. (K) BCBL1-Tet-K-RTA cells transduced with sh-Ctrl or sh-USP7 were induced with Dox (1 μg/ml) for 48 h. WCLs were collected and analyzed by immunoblotting. (TIF) [file ppat.1011943.s005.tif]

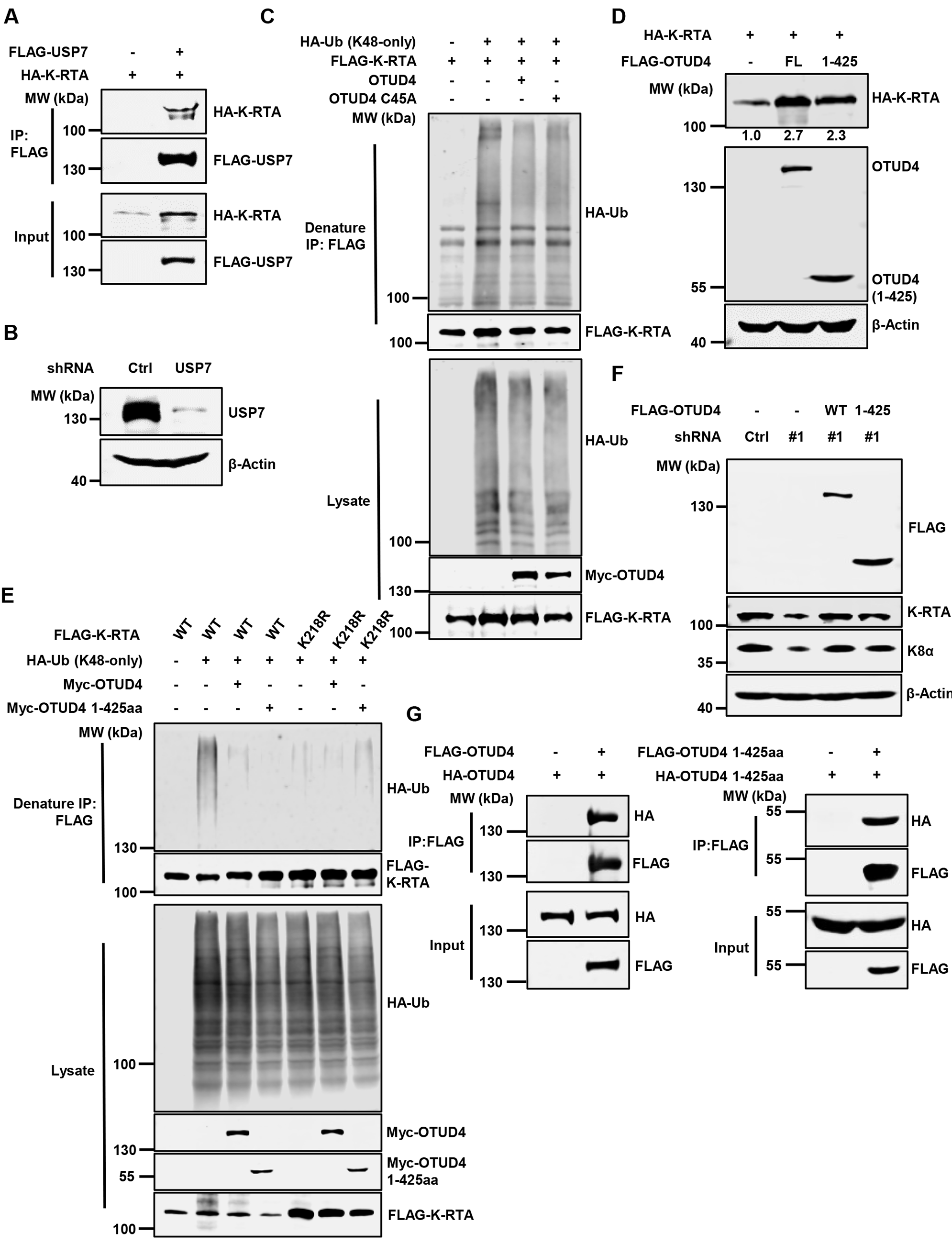

Supplement: S6 Fig — (A) WCLs were collected from HEK293T cells transfected with the indicated plasmids and subsequently subjected to immunoprecipitation with anti-FLAG affinity agarose, followed by immunoblotting. (B) Immunoblotting analysis of HEK293T cells transduced with sh-Ctrl or sh-USP7. (C) HEK293T-shUSP7 cells as described in S6B Fig. were co-transfected with FLAG-K-RTA, HA-Ub (K48-only) and Myc-OTUD4/C45A, and then treated with MG132 (10 μM). Denatured immunoprecipitation with anti-FLAG affinity agarose was performed, followed by immunoblotting. (D) HEK293T cells were co-transfected with FLAG-OTUD4 full length (FL) or 1-425aa with HA-K-RTA, followed by immunoblotting at 24 h post-transfection. (E) HEK293T cells were co-transfected with FLAG-K-RTA or FLAG-K-RTA-K218R, HA-Ub (K48-only), and Myc-OTUD4 full length or 1-425aa, and then treated with MG132 (10 μM). Denatured immunoprecipitation with anti-FLAG affinity agarose was performed, followed by immunoblotting. (F) SLK.iBAC-GFP stable cells as described in Fig 6I were induced with Dox (1 μg/ml) for 48 h, and WCLs were analyzed by immunoblotting. (G) FLAG-OTUD4 and HA-OTUD4 or FLAG-OTUD4 (1-425aa) and HA-OTUD4 (1-425aa) were co-expressed in HEK293T cells, and co-immunoprecipitation and immunoblotting were performed at 24 h post-transfection. (TIF) [file ppat.1011943.s006.tif]
