# Supplementary material for: Non-canonical regulation of the reactivation of an oncogenic herpesvirus by the OTUD4-USP7 deubiquitinases
Source: PLoS Pathog. 2024 Jan 12;20(1):e1011943. doi: 10.1371/journal.ppat.1011943 (PMC10810452; doi:10.1371/journal.ppat.1011943)
Supplement: S2 Table — (DOCX) [file ppat.1011943.s008.docx]

Supplementary Table 2 primers used in this study

| **Gene** | **Sequence** | |
| --- | --- | --- |
| KSHV RTA | CGCAATGCGTTACGTTGTTG | GCCCGGACTGTTGAATCG |
| KSHV ORF56 | CACAGATTCCCGTCAATACAAA | GTATCTTCAGTAGGCGGCAGAG |
| KSHV ORF57 | CATCCTAGAGGACTCTGT | TTGCTCGTCTTCCAGTGT |
| KSHV ORF25 | ACAGTTTATGGCACGCATAGTG | GGTTCTCTGAATCTCGTCGTGT |
| KSHV ORF26 | GCTCGAATCCAACGGATTTG | AATAGCGTGCCCCAGTTGC |
| OTUD4 | GACGGCGGGGACCAG | AGACTGAGAGTGCAATACCTGCT |
| USP7 | CCAGTGCAATGCTGAATCTGA | ACGACGACTGAACGACTTTTCAT |
| USP10 | ATTGAGTTTGGTGTCGATGAAGT | GGAGCCATAGCTTGCTTCTTTAG |
| CYLD | TTTGCGTGTGTTGAAAGTACAAT | TTCCTGCGTCACACTCTCTG |
| ATCB | GTTGTCGACGACGAGCG | GCACAGAGCCTCGCCTT |
