# Supplementary material for: Non-canonical regulation of the reactivation of an oncogenic herpesvirus by the OTUD4-USP7 deubiquitinases
Source: PLoS Pathog. 2024 Jan 12;20(1):e1011943. doi: 10.1371/journal.ppat.1011943 (PMC10810452; doi:10.1371/journal.ppat.1011943)
Supplement: Attachment — Submitted filename: Responses to Reviewer Comments.pdf [file ppat.1011943.s009.pdf]

Part I - Summary
Please use this section to discuss strengths/weaknesses of study, novelty/significance, general execution and scholarship.

Reviewer #1: In this manuscript, Wang et al found that OTUD4 is a novel binding partner of KSHV RTA and facilitates KSHV lytic replication by stabilizing RTA. Interestingly, the DUB activity of OTUD4 is not required for RTA stability, while OTUD4 acts as an adaptor protein to facilitate RTA-USP7 interaction, which removes the K48-linked ubiquitination of RTA.

Response: We sincerely thank the reviewer for her/his positive comments. We have addressed all concerns from the reviewer as detailed below. Whenever possible, experiments were performed to provide answers to the reviewer's questions.

Reviewer #2: This data intense study described a function of K-Rta-OTU4 interaction in KSHV replications. The OTU domain containing 4 (OTU4) is a Lys63-specific deubiquitinylase and negative regulators of inflammatory or innate signaling pathways via deubiquitinylation of cellular receptors. The authors comprehensively demonstrated that K-Rta binds to OTU4 during reactivation at C-terminal region, and the interaction is responsible for recruitment of USP7 to the K-Rta protein. The authors further showed that recruitment of USP7 is important for increasing the stability of K-Rta via K-Rta de-ubiquitination. Knock-down of one of the deubiquitinylases significantly impairs KSHV reactivation/replications.

Response: We sincerely thank the reviewer for her/his positive comments.

The manuscript is well-written. A significant amount of data sets demonstrated the protein interactions clearly, and experiments are logically presented. Some concerns are the specificity of the OTU4's biological activity on the viral transcription. It would be important to include some cellular genes whose promoters are not regulated by K-Rta. We expect those cellular genes will not be significantly impacted by the knock-down of OTU4 or USP7. The authors showed that K63-ubiquitinylation was not changed by the overexpression of OTU4 in total cell lysates, which raises concern if the OTU4 construct is working correctly. It is helpful to include a positive control for the experiments.

Response: We sincerely thank the reviewer for these constructive comments. We have addressed all concerns from the reviewer as detailed below. Following the reviewer's suggestions, we have included the expression of several cellular genes that were not impacted by the knockdown of OTUD4 or USP7 (Please refer the answer to question #1). Additionally, we have included Myd88, a known OTUD4 substrate, to demonstrate that OTUD4 could efficiently catalyze K63 deubiquitination of Myd88 (Please refer to the answer to question #2).

Reviewer #3: In this report, the author has identified two K-Rta binding deubiquitinases (DUBs) that play a crucial role in stabilizing K-Rta and facilitating KSHV reactivation.

Significantly, the author has demonstrated that OTUD4 utilizes its N-terminal domain to recruit both K-Rta and USP7, thereby enhancing the deubiquitination process and subsequently stabilizing K-Rta. This, in turn, results in the augmentation of herpesvirus reactivation. This discovery holds significant importance, as it sheds light on the deubiquitination in viral replication.

Response: We sincerely thank the reviewer for her/his positive and constructive comments. We have addressed all concerns from the reviewer as detailed below. Whenever possible, experiments were performed to provide answers to the reviewer's questions.

Part II – Major Issues: Key Experiments Required for Acceptance
Please use this section to detail the key new experiments or modifications of existing experiments that should be absolutely required to validate study conclusions. Generally, there should be no more than 3 such required experiments or major modifications for a "Major Revision" recommendation. If more than 3 experiments are necessary to validate the study conclusions, then you are encouraged to recommend "Reject".

Reviewer #1: Overall, the data has high quality and could support the authors' conclusion.

Response: We sincerely thank the reviewer for her/his positive comments.

Reviewer #2: 1. For Fig. 2A and Fig. 5D, please include cellular genes as comparisons to demonstrate specificity. It is important to show that knock-down of OTU4 or USP7 did not globally reduces cellular transcription activity.

Answer: We thank the reviewer for the instructive comments. We have fully taken the reviewer's suggestions. To demonstrate the specificity, we chose three host genes, *MAVS*, *ALKBH2,* and *ALKBH3*, all of which are regulated by OTUD4 at the post-transcriptional level. Indeed, our results revealed no significant transcriptional changes after knock-down of OTUD4 or USP7 (new Figure S2A and S5J), indicating that knockdown of OTUD4 or USP7 does not globally reduce cellular transcription activity.

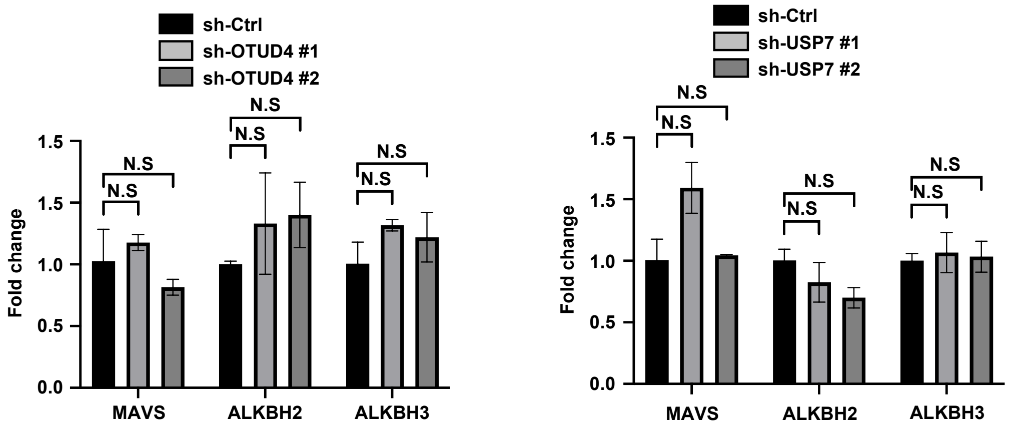

2. Figure S3 D. It is a little strange that overexpression of OTU4 did not affect amount of cellular K-63 ubiquitinylation in cotransfection study in total cell lysates. This raises a question if the OTU4 construct is O.K. It is important to include a positive control to show that the deubiquitinylation activity for both OTU4 and USP7.

Answer: We thank the reviewer for raising these critical points. OTUD4 functions as a DUB for a specific subset of host substrates, hence overexpression of OTUD4 may not substantially affect global K63 ubiquitination.

Following the reviewer's suggestions, we have chosen the known substrate of OTUD4 (Myd88) and USP7 (MDM2) to demonstrate the DUB activity of OTUD4 and USP7. Our results indicate that WT OTUD4 effectively reduced K63 ubiquitination of Myd88, while the C45A mutant showed impaired DUB activity (new Figure S3E). Additionally, USP7 clearly reduced K48 ubiquitination of MDM2 (new Figure S5F).

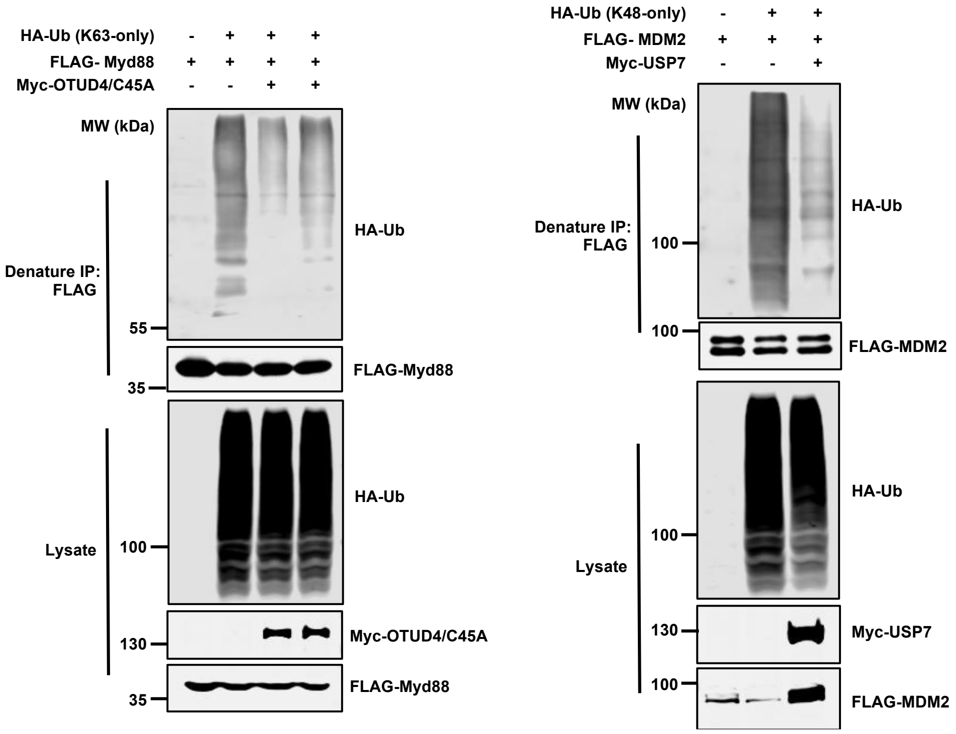

3. The OTU4 is primarily localizes cytoplasmic and K-Rta is a nuclear protein. The majority of experiments were performed with transient transfection except Fig. 4A. However, the Fig. 4A does not have input proteins in the same gel, which makes difficult

to determine how much proportion of K-Rta or OTU4 is interacting each other endogenously. The ubiqutin pathways regulates multiple cellular signaling events including transcription, thus inhibition of transcription by knock-down of the deubiquitinylase can be due to indirect mechanisms. It is therefore important to demonstrate endogenous co-localization between OTU4 and K-Rta, and may repeat the experiment with appropriate controls.

Answer: Following the reviewer's suggestions, we have repeated the immunoprecipitation experiments and have the input (0.5%) and IP bands in the same gel (new Figure 4A).

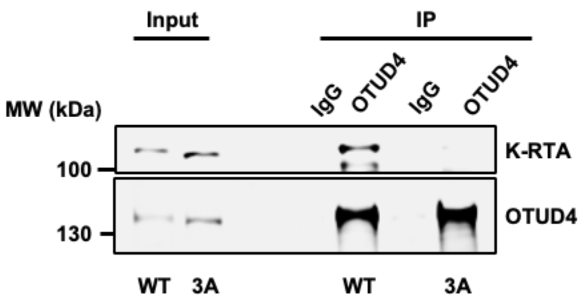

We thank the reviewer for the insightful suggestions about our immunofluorescence experiments. We completely agree with the reviewer's points.

We attempted to perform endogenous OTUD4 staining with various highly cited commercial antibodies that work well for immunoblotting (Proteintech, 25070-1-AP; Sigma, HPA036623; Abcam, ab106368). Unfortunately, these antibodies do not work for immunofluorescence staining (i.e., we could observe strong non-specific staining signals even after we effectively knocked down OTUD4 expression). So we stably expressed FLAG-OTUD4 in KSHV-latently infected cells through lentiviral transduction, since optimized lentiviral-mediated protein expression levels can be comparable to endogenous proteins, and the FLAG-tag generally does not interfere with protein localization. We repeated the experiments with appropriate controls as suggested by the reviewer, and showed that both K-RTA staining and FLAG staining were specific. We conclude that our immunofluorescence staining results indicate that OTUD4 translocates into the nucleus after KSHV lytic reactivation and co-localizes with KSHV K-RTA (new Figure 3C). However, the mechanism underlying OTUD4 translocation into the nucleus following KSHV lytic reactivation remains unknown. A plausible hypothesis is that KSHV K-RTA interacts with OTUD4, facilitating its translocation into the nucleus. We have incorporated these points raised by the reviewer into the discussion (line 332-337).

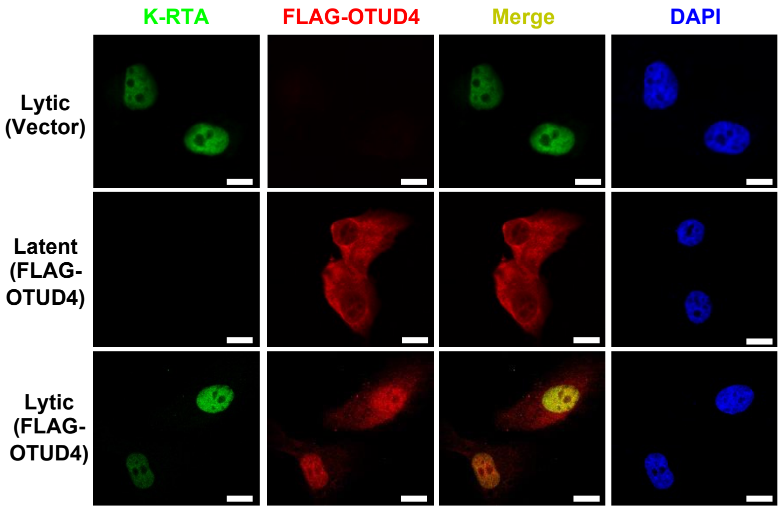

Reviewer #3: 1. The author has successfully identified the FRD motif in the C-terminal region of K-Rta that mediates its binding to the N-terminal of OTUD4. It is recommended that the author provides a more detailed description of the strategy employed for domain mapping in Figure Legend.

Answer: We appreciate the reviewer's valuable suggestions. We have provided a detailed description about the domain mapping experiments in Figure Legend (line 642-649, 762-770).

2. While the use of BAC-transfected SLK cells is okay, it would be valuable if the author could also confirm the endogenous interaction in KSHV (+) cell lines, such as BCBL-1, and reactivate it using a chemical inducer, such as HDACi.

Answer: We thank the reviewer for the insightful suggestions.

Following the reviewer's suggestions, we have confirmed the endogenous interactions between OTUD4 and USP7, as well as OTUD4 and K-RTA, in BCBL-1 cells (new Figure S1B and 5C).

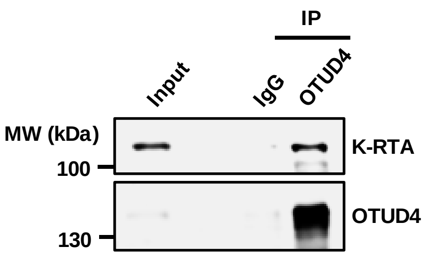

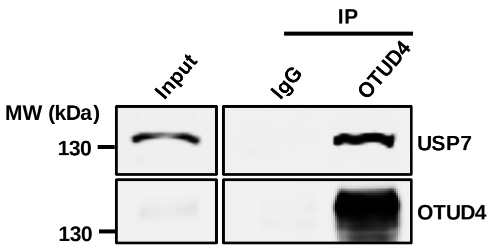

3. The identification of K-Rta K218 as the major ubiquitination site for OTUD4-USP7 is an impressive finding. The author can include it as a control.

Answer: We thank the reviewer for the insightful suggestions.

We examined the ubiquitination of K-RTA-WT and K-RTA-K218R, in the presence of OTUD4 or OTUD4 1-425aa. The results revealed that K-RTA-K218R exhibited decreased ubiquitination compared with K-RTA. Moreover, the ubiquitination of K-RTA was significantly reduced by OTUD4 and OTUD4 1-425aa, while the ubiquitination of K-RTA-

K218R remained unchanged when co-expressed with OTUD4 and OTUD4 1-425aa (new Figure S6E).

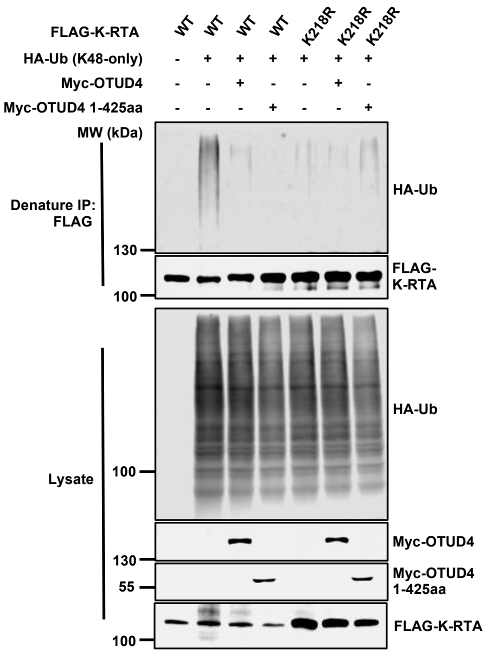

4. In Figure 4D, it is suggested that the author consider using OTUD4 overexpression, instead of knockdown, to demonstrate that RTA-3A can abolish the interaction and the function of OTUD4.

Answer: We fully take the reviewer's suggestions. Our new results indicate that the RTA-3A mutation significantly impaired progeny virion production. Additionally, OTUD4/C45A failed to promote the reactivation of KSHV-RTA-3A as it did for WT KSHV (new Figure 4D).

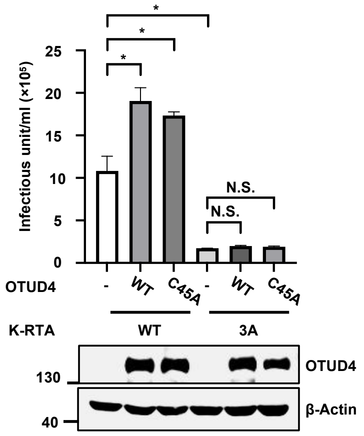

5. Again, in Figure 5C, it would be beneficial if the author could confirm the endogenous interaction in KSHV (+) cell lines, such as BCBL-1.

Answer: We have fully taken the reviewer's suggestions. Please refer to the answer to Question #2.

6. To further elucidate the role of the OTUD4-USP7 axis in regulating K-Rta ubiquitination, the author should contemplate overexpressing USP7 in both parental and OTUD4-depleted cells, in order to show the inhibitory function of USP7 in K-Rta ubiquitination.

Answer: We appreciate the insightful suggestions from the reviewer.
We performed new experiments as suggested by the reviewer, and the results indicate that USP7 failed to remove K48-linked ubiquitin chains of K-RTA in OTUD4-depleted cells (new Figure 6F).

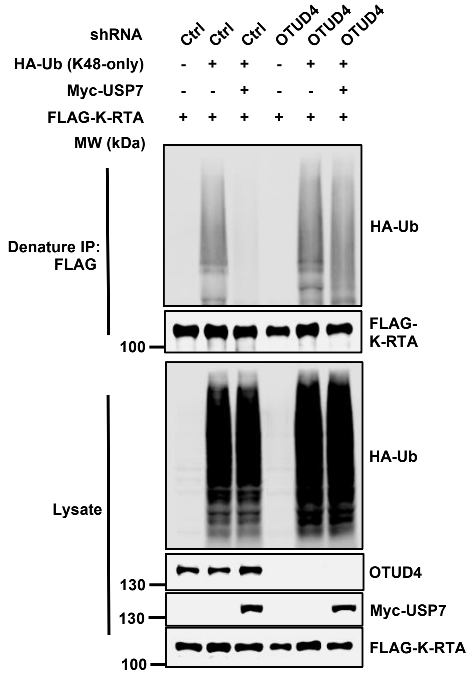

Part III – Minor Issues: Editorial and Data Presentation Modifications

Please use this section for editorial suggestions as well as relatively minor modifications of existing data that would enhance clarity.

Reviewer #1: 1. In Fig S5C right panel, it looks like that CYLD knockdown inhibits the infectious unit of KSHV.

Answer: We thank the reviewer for pointing out this.

We have repeated the experiments and our results indicate that knockdown of CYLD does not affect KSHV lytic replication (new Figure S5D).

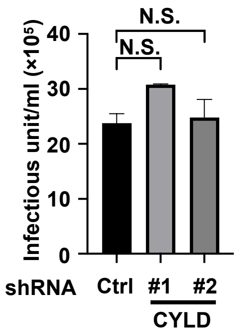

2. In Fig S6C legend, the cells used here should be HEK293T-shUSP7.

Answer: We thank the reviewer for pointing out this error. The legend for Fig S6C has been revised accordingly. (line 856).

3. Does OTUD4 or RTA has the USP7-interaction motif?

Answer: We thank the reviewer for the insightful questions.

Our domain mapping results indicate that OTUD4 (1-425aa) and the TRAF domain of USP7 mediated the interaction between the two proteins (Figure S5H and S5G). Previous studies have established that the TRAF domain of USP7 recognizes a conserved P/A/EXXS (X represents any amino acid) motif in its substrate. Currently, we are screening the P/A/EXXS motifs in OTUD4 (1-425aa) to determine whether these motifs play critical roles in USP7 binding.

Regarding KSHV RTA, we carefully inspected its sequence and found it contains a KXXXK motif (X represents any amino acid), which can be potentially recognized by the UBL domain of USP7. However, our data show that OTUD4 is required for the interaction between K-RTA and USP7 (Figure 6C), and knockdown of OTUD4 abolishes the deubiquitination of K-RTA by USP7 (new Figure 6F), indicating that K-RTA does not directly interact with USP7.

We have incorporated the points raised by the reviewer into the discussion (line 327-332).

4. Does OTUD4 also affect other herpesviruses, such as MHV68 and EBV?

Answer: We thank the reviewer for the insightful questions.

We are carrying out following studies to evaluate whether OTUD4 affect other herpesviruses, such as MHV68 and EBV. Our preliminary data indicate that OTUD4 deletion greatly inhibits MHV68 replication both in vitro and in vivo. While we speculate that OTUD4 may have a critical role in EBV replication, we are yet to generate experimental evidence. The points raised by the reviewer have been integrated into the discussion (line 365-367).

Reviewer #2: 1. It is more convincing if the authors could provide flow cytometry data for the recombination KSHV infection.

Answer: We thank the reviewer for pointing out this. We presented flow cytometry data for the recombination KSHV infection (new Fig S4B).

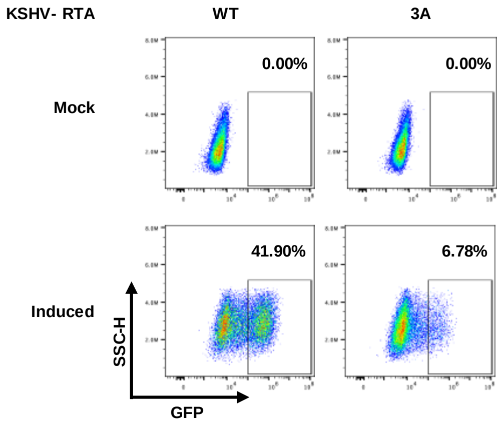

2. Line 124: it should read decreased transcription (not increased transcription).

Answer: We appreciate the reviewer’s comments and have corrected the error (line 140).

Reviewer #3: 1. For KSHV, we usually use K-Rta instead of Rta. Can the author correct it?

Answer: We thank the reviewer for the instructive comments. We have made the modifications accordingly.

2. The manuscript would be improved by undergoing English editing, but it's not mandatory.

Answer: We thank the reviewer for the instructive comments. Our manuscript has undergone professional editing by a native English speaker.
